# The first mitochondrial genome of *Parastratiosphecomyia* (Diptera: Stratiomyidae)

**DOI:** 10.1080/23802359.2020.1870886

**Published:** 2021-02-09

**Authors:** Kai Hu, Zaihua Yang

**Affiliations:** ^a^Guizhou Academy of Forestry, Guiyang, Guizhou, PR China

**Keywords:** Mitogenome, stratiomyidae, phylogenetic analysis, brachycera, *Parastratiosphecomyia szechuanensis*

## Abstract

The complete mitochondrial genome of *Parastratiosphecomyia szechuanensis* Lindner, 1954 was sequenced and analyzed in this study. The mitochondrial genome is 16,414 bp in length, including the 37 typical insect mitochondrial genes and a large control region. All PCGs end with complete termination codon TAA or TAG. Most PCGs initiated by standard start codon ATN, except for *cox1* which starts with TCG. The phylogenetic analysis based on the nucleotide sequence data of 13 PCGs recovered the monophyly of Stratiomyidae and the sister relationship between Xylomyidae and Stratiomyidae.

The superfamily Stratiomyoidea belongs to the order Diptera, and is one of the key groups connecting Nematocera and Brachycera (Yang et al. 2014). Stratiomyidae is the larger of the two families of Stratiomyoidea with 282 genera and more than 30,000 species recorded in the world, has a relatively important evolutionary status (Zhou et al. 2017). However, there are only three mitochondrial genomes of Stratiomyidae in GenBank (as of 8 September 2020). Here, we sequenced the mitochondrial genome of *Parastratiosphecomyia szechuanensis* and investigated the phylogenetic relationships within Brachycera.

The adult specimens used in this study were collected from Luodian county (E106.7917, N25.4075), Guizhou province, China by Zaihua Yang in May 2020, and genetic material of this soldier fly (GZAF-2020-DS1435) was preserved in Insect Herbarium of Guizhou Academy of Forestry, Guiyang. The identification was based on morphological Characteristics (Yang et al. 2014). Next, the genomic DNA of *P. szechuanensis* was sequenced by next-generation sequencing (NGS). The complete mitochondrial genome was assembled and annotated using MitoZ (Meng et al. 2019). Maximum-likelihood phylogenetic analysis was performed by IQ-TREE version 1.6.10 (Vienna, Austria) (Nguyen et al. 2015) using the ultrafast bootstrap (UFB) approximation approach (Minh et al. 2013) with 8000 replicates.

The complete mitochondrial genome of *P. szechuanensis* (Genbank accession no. MW039153) is double-stranded circular DNA molecule with a size of 16,414 bp. The mitochondrial genome includes the 37 typical insect mitochondrial genes (13 protein-coding genes, 22 transfer *RNA* genes, and two ribosomal *RNA* genes) and a large AT-rich region (control region). The gene order of the newly sequenced Stratiomyidae is consistent with other known Stratiomyidae (Qi et al. 2017; Zhan et al. 2020). In the mitochondrial genome, the 23 genes (nine PCGs and 14 tRNAs) were encoded by the majority strand (J-strand) while 14 genes (four PCGs, eight *tRNAs*, and two *rRNA* genes) were encoded by the minority strand (N-strand). The overall nucleotide compositions of *P. szechuanensis* are A = 39%, C = 16.3%, G = 9.5%, and T = 35.2%, indicating the mitochondrial genome has a strong AT nucleotide bias. Most PCGs (*atp6*, *atp8*, *cox2*, *cox3*, *cytb*, *nad1*, *nad2*, *nad3*, *nad4*, *nad4L*, *nad5*, and *nad6*) initiated by standard start codon ATN (ATA/T/G/C), except for *cox1* which starts with TCG. All PCGs end with complete termination codon TAA or TAG.

Phylogenetic analysis of 19 species of Diptera, including two outgroups from Nematocera, based on the concatenated dataset of 13 PCGs, yielded topology is similar to previously published phylogenies (Zhou et al. 2017; Ding and Yang 2020) (Figure 1), but we have much larger taxon sample. The monophyly of all families (Tabanidae, Xylophagidae, Asilidae, and Stratiomyidae) represented by more than one species received support. The phylogenetic relationships among Brachycera is (Rhagionidae + ((Athericidae + Tabanidae) + (Xylophagidae + ((Asilidae + Nemestrinidae) + (Stratiomyidae + Xylomyidae))))). Furthermore, the sister relationship between Xylomyidae and Stratiomyidae is recovered with the high support value (BS = 95), in agreement with previously those found based on mitochondrial genome data (Zhou et al. 2017; Ding and Yang 2020).

**Figure 1.**
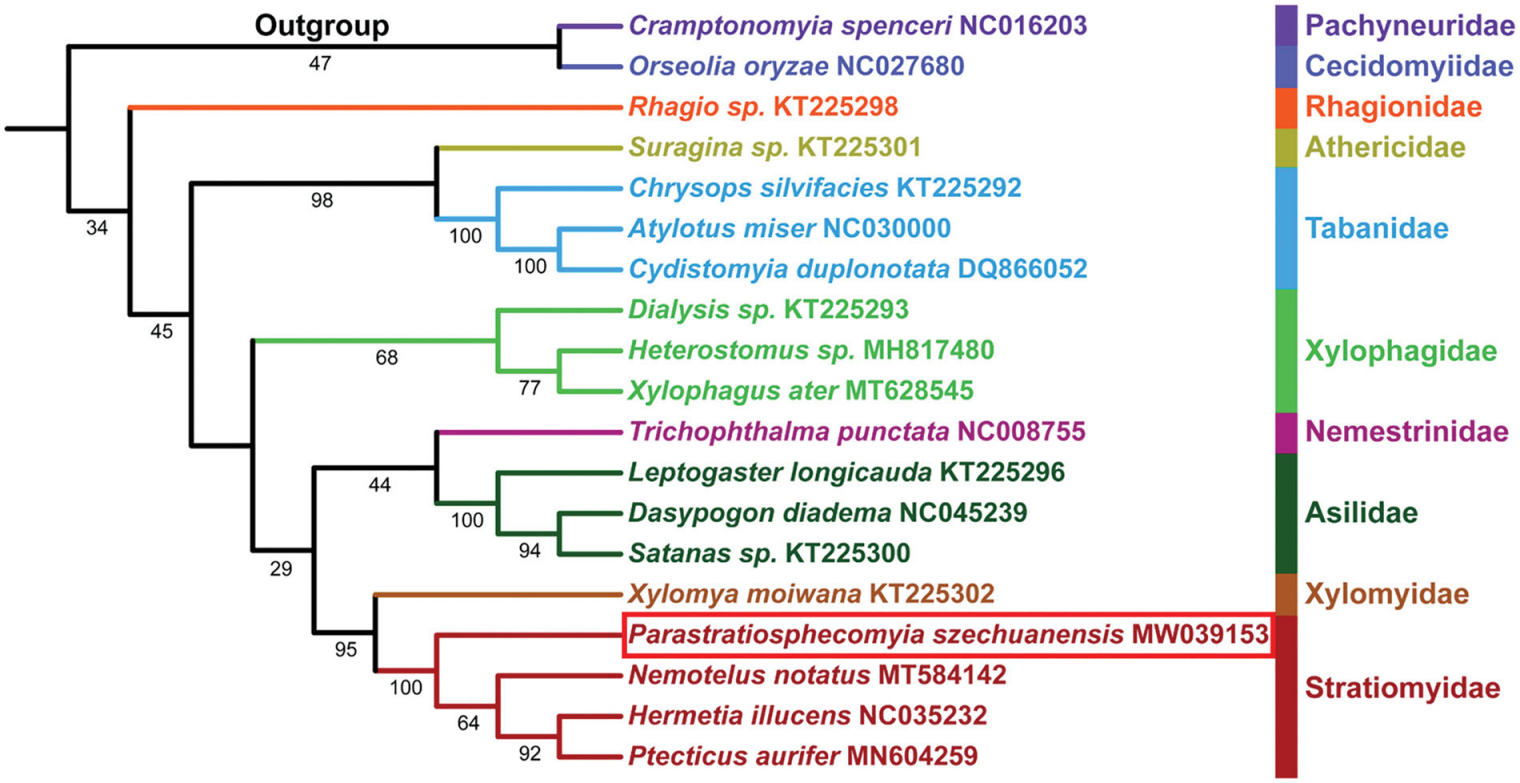
Maximum-likelihood tree reconstructed using the PCG123 dataset of *P. szechuanensis* and other 16 species belonging to eight related families of Brachycera. Two species of Nematocera were used as outgroups. Bootstrap support values (BS) are indicated on branches.

## Data Availability

The data that support the findings of this study are openly available in GenBank of NCBI at https://www.ncbi.nlm.nih.gov/, GenBank Accession Number: MW039153.
